# GD2 and GD3 gangliosides as diagnostic biomarkers for all stages and subtypes of epithelial ovarian cancer

**DOI:** 10.3389/fonc.2023.1134763

**Published:** 2023-04-14

**Authors:** Alba Galan, Arturo Papaluca, Ali Nejatie, Emad Matanes, Fouad Brahimi, Wenyong Tong, Ibrahim Yaseen Hachim, Amber Yasmeen, Euridice Carmona, Kathleen Oros Klein, Sonja Billes, Ahmed E. Dawod, Prasad Gawande, Anna Milik Jeter, Anne-Marie Mes-Masson, Celia M. T. Greenwood, Walter H. Gotlieb, H. Uri Saragovi

**Affiliations:** ^1^ Translational Cancer Center, Lady Davis Institute-Jewish General Hospital, McGill University, Montreal, QC, Canada; ^2^ Pharmacology and Therapeutics, McGill University, Montreal, QC, Canada; ^3^ Department of Ob-Gyn, Jewish General Hospital, McGill University and Segal Cancer Center, Lady Davis Institute of Medical Research, Montreal, QC, Canada; ^4^ Clinical Sciences Department, College of Medicine, University of Sharjah, Sharjah, United Arab Emirates; ^5^ Centre de recherche du Centre Hospitalier de l’Université de Montréal (CRCHUM) and Institut du Cancer de Montréal, Montreal, QC, Canada; ^6^ Division of Gynecologic Oncology, Department of Obstetrics and Gynecology, Université de Montréal, Montreal, QC, Canada; ^7^ Gerald Bronfman Department of Oncology, McGill University, Montreal, QC, and Department of Epidemiology, Biostatistics and Occupational Health, McGill University, Montreal, QC, Canada; ^8^ R&D Department, AOA Dx Inc, Cambridge, MA, United States; ^9^ Department of Medicine, Université de Montréal, Montreal, QC, Canada; ^10^ Ophthalmology and Vision Science. McGill University, Montreal, QC, Canada

**Keywords:** tumor marker, diagnostic test, cancer screening, ovarian cancer, ELISA, immunohistochemistry, ganglioside, liquid and tissue biopsy

## Abstract

**Background:**

Ovarian cancer (OC) is the deadliest gynecological cancer, often diagnosed at advanced stages. A fast and accurate diagnostic method for early-stage OC is needed. The tumor marker gangliosides, GD2 and GD3, exhibit properties that make them ideal potential diagnostic biomarkers, but they have never before been quantified in OC. We investigated the diagnostic utility of GD2 and GD3 for diagnosis of all subtypes and stages of OC.

**Methods:**

This retrospective study evaluated GD2 and GD3 expression in biobanked tissue and serum samples from patients with invasive epithelial OC, healthy donors, non-malignant gynecological conditions, and other cancers. GD2 and GD3 levels were evaluated in tissue samples by immunohistochemistry (n=299) and in two cohorts of serum samples by quantitative ELISA. A discovery cohort (n=379) showed feasibility of GD2 and GD3 quantitative ELISA for diagnosing OC, and a subsequent model cohort (n=200) was used to train and cross-validate a diagnostic model.

**Results:**

GD2 and GD3 were expressed in tissues of all OC subtypes and FIGO stages but not in surrounding healthy tissue or other controls. In serum, GD2 and GD3 were elevated in patients with OC. A diagnostic model that included serum levels of GD2+GD3+age was superior to the standard of care (CA125, p<0.001) in diagnosing OC and early-stage (I/II) OC.

**Conclusion:**

GD2 and GD3 expression was associated with high rates of selectivity and specificity for OC. A diagnostic model combining GD2 and GD3 quantification in serum had diagnostic power for all subtypes and all stages of OC, including early stage. Further research exploring the utility of GD2 and GD3 for diagnosis of OC is warranted.

## 1 Introduction

Ovarian cancer (OC) is the most lethal gynecologic cancer and accounts for an estimated 239,000 new cases and 152,000 deaths worldwide each year (1). The current 5-year survival rate is <50%, and 15% of patients die within 2 months of diagnosis. The high mortality rate is in part related to lack of effective diagnostics because delays in diagnosis consequently delay therapeutic intervention (2, 3).

Although OC is often labeled as a silent killer, 95% of all OC patients experience symptoms for many months prior to diagnosis (4, 5). Indeed, 72% of patients with high-grade serous OC exhibit symptoms at early FIGO stages (6), and 84% consult with a doctor (2). Despite the presence of symptoms, the average delay in receiving a diagnosis is 9 months (2, 7). A delay in the diagnosis of as little as 3 months has been shown to allow cancer progression (3, 5, 8, 9) and to reduce 5-year overall survival (10).

Despite advances in treatment of OC, there continues to be a lack of early and effective diagnostic tools (11). The diagnosis of OC is commonly made using a combination of imaging (pelvic ultrasonography), tumor markers, and morphological and clinical findings. Tumor biomarkers used to aid in diagnosis include cancer antigen 125 (CA125) and human epididymis protein 4 (HE4) (4, 5, 12). However, there are currently no tumor markers that are completely specific, and all diagnostics are inadequate at detecting early-stage OC (2). Thus, the majority of women are diagnosed at late stages, and long-term OC survival rates remain low (2, 13). Improved diagnostic tools are necessary to enable earlier diagnosis and earlier treatment, which is expected to reduce morbidity and mortality, improve quality of life, and reduce health care costs.

Gangliosides are a class of sialic-acid-containing glycolipids that are expressed in plasma membranes of nearly all vertebrate cells. The GD2 and GD3 gangliosides are unique from other gangliosides in that their expression is low/absent in normal cells but high in tumor cells (14–16). GD2 and GD3 are etiological to cancer onset or progression (14, 16, 17) and cause immune suppression, allowing tumors to evade immune responses (14, 18). These features make GD2 and GD3 suitable targets for cancer therapy, and anti-ganglioside therapeutics is an expanding field.

In addition, GD2 and GD3 are shed into the extracellular environment (19–22) and may be measured in blood, which is easier to obtain than tissue biopsies. Biomarkers with these characteristics are preferred over surrogate markers such as CA125 or HE4 that are not etiological or persistent. Despite having characteristics that make them ideal biomarkers for diagnostic tests, GD2 or GD3 has not yet been explored as diagnostic markers in OC (14, 23). Additionally, the means to identify GD2- or GD3-expressing patients would facilitate the clinical use of anti-ganglioside therapeutics by selection of target-expressing patients.

The purpose of our study was to characterize the expression of GD2 and GD3 in the OC tissue and serum and to develop and validate a method to quantify GD2 and GD3 in serum. We then applied this method to develop an algorithm that would allow for the diagnosis of multiple OC subtypes and FIGO stages, including the hard-to-diagnose early stages I/II and low CA125 population.

## 2 Materials and methods

### 2.1 Tissue and blood samples

**Figure 1:**
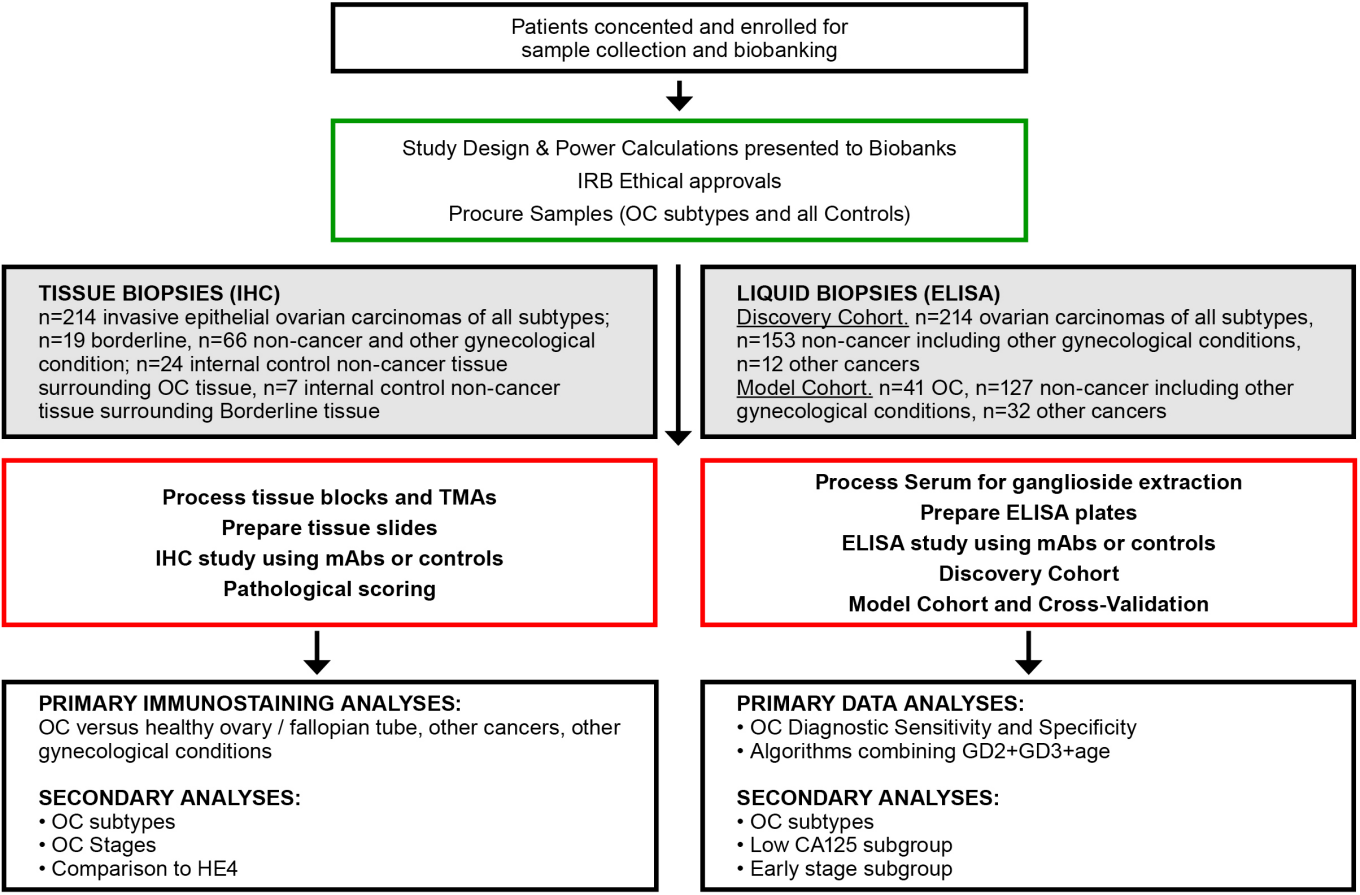
Flowchart of experimental approaches and analyses. OC, ovarian cancer; IHC, immunohistochemistry; TMA, tissue microarray; mAb, monoclonal antibody; ELISA, enzyme linked immunosorbent assay.

Tissue and serum samples were procured from biobank sources (see flowchart of the study in **Figure 1**). No individually identifiable data were used, and ethical approval was obtained from the Institutional Review Boards at each biobank: Jewish General Hospital (JGH, Montreal, QC, Canada, Protocol #15-070), the Centre de recherche du Centre hospitalier de l’Université de Montréal (CRCHUM, Montreal, QC, Canada, Protocol #BD04.002), and the commercial biobank BioIVT (Westbury, NY, USA). Informed consent was obtained from all individuals by the respective institution prior to specimen collection.

Tissue biopsy samples were collected at scheduled surgery, and all were treatment-free except for the neoadjuvant therapy (NACT) group, which received neoadjuvant chemotherapy prior to surgery. Tissue samples for immunohistochemistry were procured from JGH (n=212) and CRCHUM (n=87) for a total of N=299 samples.

Serum samples in the discovery cohort were procured from three biobanks. Samples that were procured from JGH (n=119) and CRCHUM (n=200) were not case-controlled, as there were not sufficient healthy donor samples. Healthy controls (n=60) were procured from BioIVT (total N=379) (**Supplementary Table S2**). The model cohort (n=200) included high-quality case-controlled samples obtained from BioIVT. The model cohort only included samples of sufficient quality as defined by a documented storage age of <5 years at −80°C, and a documented maximum of one freeze–thaw cycle, to minimize sample degradation due to age and multiple freeze–thaw cycles. Serum samples were excluded if they were icteric, lipemic, and hemolytic, and had substantial particulates. Serum and tissue samples with insufficient clinical data were also excluded. Serum CA125 and HE4 levels (when available) and other clinical characteristics were obtained from clinical charts. Menopausal status was unknown for many patients; therefore, a cutoff of ≥50 years of age was used as a surrogate for post-menopausal status.

### 2.2 Antibodies

For the immunohistochemistry (IHC) tissue, anti-GD2 14G2a (BD Pharmingen, Cat. 554272, used at 1:400 or 1:1200 depending on lot), anti-GD3 R24 (Abcam, Cat. ab11779, used at 1:400 or 1:200 depending on lot), and anti-human HE4/WFDC2 antibody (R&D systems, MAB6274, used at 1:500) were used. The IHC primary incubation was overnight at 4°C, followed by washing and incubation with secondary reagents for 1–2 h at room temperature. We developed anti-GD2 mAb Clone 19 and anti-GD3 mAb Clone 6 for use in quantitative enzyme linked immunosorbent assay (ELISA) evaluation of serum (characterized in **Supplementary Figure S6** and reference (17)). Secondary reagents were as follows. For flow cytometry, anti-mouse IgG conjugated to fluorescein (BD Bioscience, Cat. 554011). For ELISA, anti-mouse IgG conjugated to horseradish peroxidase (HRP) (Sigma, Cat. A0168, used at 1:1,000). For IHC, anti-mouse IgG coupled to horseradish peroxidase (HRP) (Vector Laboratories, ZF0718, used at 1:2,000).

### 2.3 Tissue sample immunohistochemistry

Tissue blocks (paraffin-embedded blocks) and microarrays were procured, and duplicated cores for each sample were studied. The tissue microarrays contained multiple OC subtypes, control healthy fallopian tube, healthy ovarian tissue, and tissue from non-malignant gynecological conditions. A summary of the samples is provided in **Supplementary Table S1**.

For tissue blocks, paraffin-embedded 4-µm-thick tissue sections were deparaffinized and washed in phosphate-buffered saline (PBS). For tissue microarrays and block tissue, endogenous hydrogen peroxidase and biotin were blocked with 0.3% (v/v) H_2_O_2_ and avidin/biotin blocking kit (Vector Laboratories, Cat. SP-2001), respectively. Unspecific background was blocked with blocking reagent (Vector Laboratories, BMK-2202) followed by overnight incubation with mouse-anti-GD2 mAb or mouse-anti-GD3 primary mAbs at 4°C. Sections were incubated with biotinylated anti-mouse IgG followed by streptavidin coupled to horseradish peroxidase (HRP) (Vector Laboratories, ZF0718), and reactivity was revealed by DAB reaction (Vector Laboratories, SK-4105) and counterstaining with hematoxylin/eosin (Vector Laboratories, H-3502). Sections without primary antibody were used as negative control. Images were taken using a Leica ScanScope AT turbo light microscope scanner.

**Figure 2:**
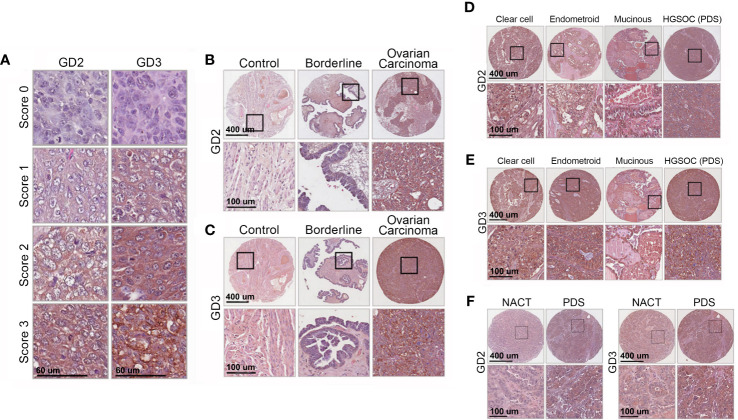
Immunohistochemistry shows high GD2 and GD3 in tissues of all OC subtypes. **(A)** Immunohistochemical detection of GD2 and GD3 in OC biopsies. Images show representative pictures of anti-GD2 and anti-GD3 antibody staining, scored as “0” (no staining), “1” (weak staining), “2” (moderate staining), and “3” (strong staining). Scores “0” and “1” were considered negative, and scores “2” and “3” were deemed positive. **(B, C)** Representative images showing GD2 and GD3 immunohistochemistry in normal, borderline ovarian tumor, and OC tissue biopsies. The bottom panels show a higher magnification of tissue within the black boxes (scale bars indicated). **(D, E)** Representative images showing GD2 and GD3 immunohistochemistry in clear cell, endometrioid, and mucinous cancer tissue biopsies, and in primary debulking surgery (PDS) cancer tissue biopsies from high-grade serous cancer (HGSOC) patients. The bottom panels show a higher magnification of tissue within the black boxes (scale bars indicated). (**F**) GD2 and GD3 immunohistochemistry in HGSOC patients with PDS or treated with neoadjuvant therapy (NACT) The bottom panels show a higher magnification of tissue within black boxes (scale bars indicated). See **Table 1** for statistical comparisons and summary data.

The tissue from single biopsy blocks was verified pathologically to contain both tumor and healthy tissue (internal control) on the same slide. The immunoreactivity of GD2 and GD3 were reviewed and scored by blinded independent readers (one a certified pathologist) using a semi-quantitative method (24). The independent researchers had a coefficient correlation= 0.70 for GD2 and 0.77 for GD3. Samples were classified according to their intensity: no immunoreactivity (0), 1+ (weak stain), 2+ (stain), and 3+ (strong stain). Scores of 0 or 1+ were considered negative, and scores of 2+ and 3+ were considered positive (**Figure 2A**). The same scoring method was used for HE4.

### 2.4 Extraction of GD2 and GD3 from human serum

Gangliosides GD2 and GD3 were extracted from serum samples following modifications of a described method (18). Briefly, 100 µl of human serum was mixed with 500 µl extraction buffer of chloroform–methanol–water with a ratio of 4:8:3, followed by vigorous vortexing. The sample was centrifuged (3,000*g* for 20 min at 4°C) into aqueous and organic phases, and the aqueous phase (range between 200 and 400 µl) was transferred into a clean tube. After adding sterile water to the collected aqueous phase (for a final ratio of chloroform–methanol–water of 4:8:5.6), a second extraction was performed by repeating the steps above. Organic solvents were removed from the samples under nitrogen gas and resuspended in ethanol.

### 2.5 Indirect enzyme-linked immunosorbent assay

Enzyme-linked immunosorbent assay (ELISA) methods were modified from a previous report (17). After isolation of glycolipids by extraction, samples (10 µl/well) were immobilized onto Clear Flat-Bottom Immuno non-sterile 96-well plates (Thermo Scientific Cat. 3455 Lot X1530419), blocked for 1 h with blocking buffer (PBS+0.1% BSA) followed by three washes with 1× PBS. Wells were then incubated with primary antibodies anti-GD2 or anti-GD3 mAbs (50 µl, 1.7 nM in blocking buffer) or negative control mouse IgG. After 1 h, the wells were washed three times with 1× PBS and then incubated with horseradish peroxidase (HRP)-conjugated anti-mouse IgG secondary antibody (50 μl, 0.06 nM in blocking buffer, Sigma, Cat. A0168) for 1 h. After three washes with 1× PBS, the colorimetric reaction was visualized with 3,3′,5,5′-tetramethylbenzidine substrate solution (TMB, Sigma, Cat. 34028), and the absorbance were read at 450 nm. All tests were performed three independent times for each extracted serum, with each sample in duplicate wells. Each plate had an internal control standard curve of GD2 (Advanced Immunochemical Inc., Cat. 9-IG6-h) or standard curve of GD3 (Avanti Polar Lipids Inc., Cat. 860060) ranging from 0 to 10 ng/well. Background sample controls include non-cancer healthy donors. Background plate controls omitted primary mAb, but with all other reagents added in the proper sequence.

### 2.6 Flow cytometry

Flow cytometry was used to characterize binding activity of proprietary mAbs manufactured in-house for ELISA immunoassays. A total of 2×10^5^ cells of EL4-GD2+ (EL4 cells expressing surface GD2 but no GD3) and EL4-GD3+ (EL4 cells expressing surface GD3 but no GD2) were studied in binding assays, as described previously (17). Negative control Jurkat and R1.1. cell lines were used, as they do not express GD2 or GD3 but express GM1 and other gangliosides. Cells were incubated for 20 min on ice with positive control anti-GD2 mAb or anti-GD3 mAb (each at 13 nM), or control IgG, followed by fluorescein-conjugated anti-mouse IgG secondary (1.8 nM, Sigma). Cells were assessed in a flow cytometer (Becton-Dickinson) and data analyzed using CellQuest software.

### 2.7 Quantification of CA125 and HE4 from human serum

CA125 concentrations were available from clinical charts. Where CA125 concentrations were not available, CA125 were quantified using the R&D Systems/Protein Simple Instrument and the Simple Plex Human CA125/MUC16 Cartridge SPCKB-PS-000475. HE4 concentrations were quantified using R&D Systems/Protein Simple Instrument and Simple Plex Human HE4/WFDC2 Cartridge SPCKB-PS-000542).

### 2.8 Statistical analysis

The association of GD2 and GD3 expression with clinicopathological parameters was analyzed using one-sided Kruskal–Wallis test with two-sided Tukey test with significance set at p<0.05. One-sided Kruskal–Wallis test was used for multiple comparisons to calculate significance among groups. If Kruskal–Wallis test showed significance, a two-sided Tukey test was done to evaluate significance of specific groups. Two-sided Mann–Whitney U test was used to perform the analysis between two groups. A p-value of <0.05 was considered statistically significant. Statistical differences were calculated using Python 3.8, scipy 1.9.1, scikit-posthocs 0.7.0. Box plots were generated using GraphPad Prism version 8.0.0 for Windows, GraphPad Software, San Diego, CA, USA.

### 2.9 Model cohort power analyses for model building (receiver operating characteristic)

Our performance goal was to achieve sensitivity of 97%. A two-sided power calculation was used to determine the number of cases needed with a minimum effect of 80%, power of 80%, and confidence of 95%. The analysis indicated that 41 confirmed OC samples by histopathology would achieve these targets. Considering a 20% prevalence rate of disease, the total number of individual serum samples needed for developing a model was 200, of which 41 are samples from OC subjects confirmed by histopathology and the rest were controls. Power calculations were performed using Python 3.8, statsmodels 0.12.2.

### 2.10 Model cohort receiver operating characteristic curve analysis

Receiver operating characteristic (ROC) curves were constructed using Python 3.8, Sklearn 0.24.2 for modeling, statsmodels 0.12.2 for power analysis, scikit posthocs 0.7.0, and scipy 1.9.1 for statistical tests to evaluate the diagnostic performance of the biomarkers GD2 and GD3 and to compare with the performance of standard of care biomarkers CA125 and HE4 in ELISA assays. Logistic regression models were estimated for each marker individually and for panels of markers to differentiate between patients with and without OC. For each logistic regression model, ROC curves were constructed, and the area under the curve (AUC-ROC) was compared between two markers or panels of markers using a non-parametric method, which accounts for the correlation induced through the measurement of the two panels on the same set of patients. The area under the curve (ROC-AUC) was calculated with a 95% confidence interval. The model dataset included 200 samples, and the model was cross-validated using a k-fold method. In the overall cohort, the data were randomly divided into fivefold, maintaining a consistent case–control ratio in each subset. In the early-stage (FIGO I/II) cohort, the data were randomly divided into threefold to increase coverage of underrepresented samples. Logistic models were fit leaving out one subset in turn, and performance was evaluated in the samples left out. Multiple training iterations were performed training on k folds and testing on the last one changing the test fold in each iteration. This was repeated, leaving out each group in turn. For cross-validation experiments, an average score for each fold iteration was obtained to calculate an average AUC for each model. Performing a 1,000 k-fold cross-validation tested model stability. The ROC curves produced by the 1,000 k-fold cross-validation confirmed model stability when compared to the reported AUCs. Cross-validation controls the upward bias in estimating operating characteristics of the logistic regression model on the same set of patients from which the model was initially fitted. Discrete cutoffs for each biomarker were not used. The biomarkers were used as continuous variables in the univariate and multivariate logistic regression models, where the binary outcome being presence or absence of OC. For each logistic regression model, a model constant was determined and a weighted coefficient of each variable, which calculated a predicted probability for each patient using each of the logistic regression models, the resulting predicted probability values ranging from 0 to 1 for each model. A test result was considered negative if the predicted probability was less than a selected threshold and positive if it was greater than or equal to a selected threshold. The sensitivity and specificity for all possible predicted probability values (i.e., from 0% to 100%) were determined for each model, and the specificity at 97% sensitivity was reported. The two-sided DeLong method was used to calculate 95% CI and the difference between ROC-AUC curves (25). Sensitivity and specificity were calculated for the overall cohort (n=200) and an early-stage cohort (n=173). Confidence intervals for sensitivity, specificity, and accuracy are used the Clopper–Pearson exact method. p-values were calculated using a two-sided Fisher’s exact test. For all statistical comparisons, a p-value of <0.05 was considered statistically significant.

## 3 Results

### 3.1 Immunohistochemical detection of GD2 and GD3 in ovarian cancer

A total of 299 different tissue samples (tissue blocks + microarrays) were evaluated for GD2 and GD3 expression (mean patient age, 59 ± 12.4 years; range, 25–91 years) (**Table 1**). Clinical characteristics of tissue samples used for IHC is presented in **Supplementary Table S1**. Among tissue block sections, GD2 and GD3 expression was identified in OC tissues (71% positive for GD2 and 63% positive for GD3). In all positive tissue block samples, GD2 and GD3 staining was restricted to tumor cells, whereas the healthy surrounding tissue was negative (**Figures 2B, C**). In addition, GD2 and GD3 staining was homogeneously distributed in the plasma membrane and in cytoplasmic organelles. Control tissue sections (e.g., healthy fallopian tube, healthy ovary sections, healthy endometrium) did not stain for GD2 or GD3 (**Figures 2B, C**). Analysis of tissue microarray IHC staining yielded similar results (**Figures 2D–F**). GD2 was detected in 78% of OC tissues, and GD3 was detected in 80% of OC tissues as single positive. Overall, 88% of OC tissue samples were positive for either GD2 or GD3.

**Table 1:** Summary of immunohistochemistry analysis of GD2 and GD3 expression in combined analysis of tissue biopsy block sections and microarrays.

Parameters	GD2+ expression in tissue			GD3+ expression in tissue		
	Number (positive/total)	% positive	p-value *vs*. control tissue	Number (positive/total)	% positive	p-value *vs*. control tissue
Diagnosis						
**Ovarian Carcinoma (OC)**	(166/214)	78	**<0.001**	(167/214)	78	**<0.001**
HGSOC-PDS	(56/68)	82		(56/68)	82	
HGSOC-NACT	(34/52)	65		(33/52)	63	
Clear cell	(29/40)	73		(33/40)	83	
Endometroid	(33/38)	87		(30/38)	79	
Mucinous	(14/16)	88		(15/16)	94	
**Borderline ovarian tumor**	(9/19)	47	**0.001**	(8/19)	42	**<0.001**
**Total control tissues**	(1/97)	1		(1/97)	1	
**Internal control (from OC patients)**	(0/31)	0		(0/31)	0	
Non-cancer surrounding OC tumors	(0/24)	0		(0/24)	0	
Non-cancer surrounding borderline tumors	(0/7)	0		(0/7)	0	
**External control (from other patients)**	(1/66)	2		(1/66)	2	
Benign gyn condition ovary	(1/11)	9		(1/11)	9	
Normal ovary	(0/24)	0		(0/24)	0	
Normal fallopian tube	(0/9)	0		(0/9)	0	
Normal tissue (organ not documented)	(0/7)	0		(0/7)	0	
Normal endometrial tissue	(0/15)	0		(0/15)	0	
OC FIGO stage						
I	(42/54)	78	**<0.001**	(44/54)	81	**<0.001**
II	(18/21)	86	**<0.001**	(17/21)	81	**<0.001**
III	(87/118)	74	**<0.001**	(87/118)	74	**<0.001**
IV	(16/18)	89	**<0.001**	(16/18)	89	**<0.001**
Undefined stage	(3/3)	100	N/A	(3/3)	100	N/A
**Primary treatment**			p-value *vs* NACT			p-value *vs* NACT
PDS	(56/68)	82	**<0.001**	(56/68)	82	**<0.001**
NACT	(34/52)	65		(33/52)	63	
**Menopausal status**			p-value *vs* age <50			p-value *vs* age <50
Ovarian carcinoma						
Post-menopause (age ≥50 years)	(135/172)	78	0.125	(138/172)	80	0.068
Pre-menopause (age <50 years)	(31/42)	74		(29/42)	69	
Borderline ovarian tumor						
Post-menopause (age ≥50 years)	(7/14)	50	0.479	(6/14)	43	0.437
Pre-menopause (age <50 years)	(2/5)	40		(2/5)	40	

Final GD2 or GD3 scores are shown as a percentage (%) of samples. The total number of tissue samples (N=299) consisted of OC (n=214), borderline ovarian tumor (n=19), and control tissue (n=97). Controls included two types of normal tissues. One is “internal control” and consists of healthy tissue surrounding the OC tumor on block slides from OC patients (n=31). Another is “external control” and consists of cancer-free tissue from non-OC donors (n=66). Statististical significance (p<0.05) versus the indicated comparator is highlighted by bold p-values.

A combined primary analysis was conducted for all IHC tissue (blocks and microarrays) (**Table 1**). GD2 or GD3 was present in 78% of OC tissues (p<0.001 *vs*. control). There was no statistically significant effect of menopausal status (defined as age ≥50 or <50 years) on GD2 and GD3 expression for OC or borderline ovarian tumor. Notably, fallopian tubes that were free of OC were also negative for GD2 and GD3 staining.

A secondary analysis segregated IHC tissue data by OC subtype. Expression of GD2 and GD3 was observed in all OC subtypes compared to the control tissue (**Table 1**; **Supplementary Figure S1**). There were statistically significant differences between control and all OC subtypes: high-grade serous ovarian cancer (HGSOC), clear cell, endometrioid, and mucinous (all p<0.001 for GD2 and GD3 *vs*. control) (**Supplementary Figure S1**). There were no differences in GD2 or GD3 expression between OC subtypes.

Another secondary analysis evaluated differences in GD2 and GD3 by FIGO stage. Compared to controls, GD2 or GD3 was significantly elevated in all FIGO stages, including stage I (p<0.001) (**Table 1**). There were no statistical differences among the FIGO stages. Additionally, there was a significant difference in GD2 and GD3 staining scores between OC and borderline ovarian tumors (GD2, p=0.001; GD3, p<0.001) (**Supplementary Figures S1A, B**).

Within the HGSOC cohort (representing an aggressive histological subtype), data were segregated for patients who received NACT as compared to patients who had primary debulking surgery (PDS). NACT had a significantly lower (63%–65%) GD2 or GD3 positive staining compared to 82% in PDS (GD2, p=0.034; GD3, p=0.020) (**Table 1**). Moreover, NACT biopsies had lower staining intensity for GD2 or GD3 compared to PDS (both p<0.01) (**Supplementary Figures 1E, F**; **Figure 2F**).

Tissue block sections and tissue microarrays were also evaluated for HE4 immunostaining (**Supplementary Figure S2**). Expression of HE4 was positive in 83% of OC tissue samples (**Supplementary Figure S2C**). Among OC subtypes, HE4 was elevated in HGSOC, clear cell, and endometrioid (all p<0.001 *vs*. control) but was significantly lower in the mucinous subtype (**Supplementary Figure S2D**). HE4 staining also spared normal ovaries, but positive staining was observed in normal fallopian tubes (**Supplementary Figure S2H**). HE4 was significantly elevated only for stages II–IV (p=0.03), was not detected in 30% of stage I cases, and had lower statistical significance compared to GD2 or GD3 (**Supplementary Figure S2F**).

### 3.2 Quantitative ELISA of serum in the discovery cohort showed elevated GD2 and GD3 in all stages and subtypes of ovarian cancer

As primary analyses, ELISA quantification of GD2 and GD3 in the discovery cohort revealed a statistically significant increase in serum levels of GD2 and GD3 in OC compared to healthy controls (both p<0.001) (**Supplementary Figures S3A, C**).

Three subanalyses were done. Subanalysis by OC subtypes revealed that both GD2 and GD3 were significantly elevated in the serum of all invasive OC subtypes compared to healthy donors (both p<0.001) (**Supplementary Figures S3B, D**). Subanalysis of all OC with low levels of CA125 (defined as <35 U/ml at clinical diagnosis, regardless of subtype and stage) showed that GD2 and GD3 were significantly higher compared to healthy donors (p<0.001) (**Supplementary Figures S4A, C**). Lastly, subanalysis according to OC FIGO stages showed that both GD2 and GD3 were elevated in the serum of all OC stages compared to the control (all p<0.001). Comparisons of OC FIGO stage to healthy donors were statistically significant for GD2 (FIGO stage I, p=0.001; stage II, p=0.001; stage III, p=0.096; stage IV, p=0.001) and for GD3 (p=0.001 for all stages) (**Supplementary Figures S4B, D**).

For specificity controls, in addition to comparing versus healthy donors (**Supplementary Figures S3A, C**), we evaluated sera from subjects with non-gynecological conditions most likely to be suspected of OC based on symptoms or that can be positive for CA125, as they may be represented in the populations in need of diagnostics. There was no difference in GD2 or GD3 expression between these specificity controls and healthy donors (**Supplementary Figures S5A, B**), and GD2 and GD3 expression was low in both groups.

### 3.3 Quantitative ELISA of serum in the model cohort showed elevated GD2 and GD3 in ovarian cancer

**Figure 3:**
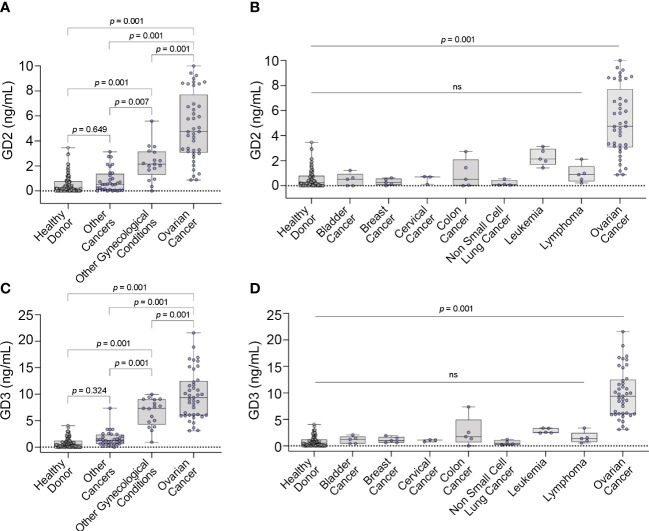
GD2 and GD3 expression in serum. Box plots displaying concentrations of **(A)** GD2 and **(C)** GD3 versus different controls. Box plots displaying concentrations of **(B)** GD2 and **(D)** GD3 with other cancers by cancer type. Box plots show median, upper and lower quartiles, and max/min values. Dots represent individual values. ns, not significant.

ELISA quantification of GD2 and GD3 in samples from the model cohort showed statistically significant elevation of GD2 and GD3 in OC compared to the control (p<0.001, **Figure 3**; **Supplementary Table S3**). Given that quantitative differences in GD2 and GD3 were observed between the discovery *vs*. model cohorts (p<0.001 for GD2 and GD3), the data from both cohorts were not combined into a single analysis. Only the data from the model cohort was used for predictive modeling.

In the primary analysis of the model cohort, there was a statistically significant overall difference between the groups for both GD2 and GD3 (both p<0.001) (**Figure 3**). There were statistically significant higher levels of GD2 and GD3 in OC samples compared to all the other groups (e.g., healthy donor, other cancers, and other gynecological conditions) (**Figure 3**). Additionally, there was a statistically significant elevation of GD2 and GD3 levels in the “other (non-malignant) gynecological conditions” group *vs*. healthy donor control, but GD2 and GD3 levels remained statistically significant *vs*. OC levels (**Figures 3A, C**). Notably, there were statistically significant higher levels of GD2 and GD3 in OC compared to all other cancer types (**Figures 3B, D**).

### 3.4 A diagnostic algorithm combining GD2 and GD3 with CA125, HE4, and/or age

We trained several different predictive models for binary outcomes, including random forest, decision trees, and k-nearest neighbors on the data from the model cohort (n=200), including unbalanced class weighting in the latter two methods. Logistic regression offered the best area under the curve (AUC) in training data and was used thereafter on receiver operator curve (ROC) analysis and cross-validation. The models included GD2, GD3, CA125, HE4, and/or age as predictors in various combinations; and the AUC was calculated for each model to quantify OC detection performance for each analyte independently and in combinations (**Table 2**).

**Table 2:** Summary and statistics of ELISA analysis—diagnostic model and cross-validation.

Overall predictive model (N=200)						
	Predictive model AUC	95% CI	p-value *vs*. CA125	5× cross-validation AUC	95% CI	p-value *vs*. CA125
CA125	0.876	0.823 – 0.942		0.877	0.777-0.977	
HE4	0.903	0.858 – 0.947	0.208	0.904	0.884 – 0.924	0.083
GD2	0.957	0.928 – 0.985	**0.010**	0.952	0.922 – 0.982	**0.032**
GD3	0.965	0.944 – 0.987	**0.001**	0.963	0.943 – 0.983	**<0.001**
GD2, GD3, age	0.988	0.977 – 0.998	**<0.001**	0.976	0.956 – 0.996	**0.002**
Early-stage predictive model (N=173)						
	**Predictive model AUC**	**95% CI**	**p-value *vs*. CA125**	**3× cross-validation AUC**	**95% CI**	**p-value *vs*. CA125**
**CA125**	0.801	0.675 – 0.939		0.773	0.613 – 0.933	
**HE4**	0.888	0.813 – 0.964	0.059	0.898	0.838 – 0.958	0.073
**GD2**	0.952	0.889 – 1.000	**0.010**	0.952	0.922 – 0.982	**0.033**
**GD3**	0.967	0.936 – 0.997	**0.014**	0.973	0.963 – 0.983	**0.043**
**GD2, GD3, age**	0.988	0.971 – 1.000	**0.002**	0.979	0.969 – 0.989	**0.006**

Statististical significance (p<0.05) indicated by bold p-values.

**Figure 4:**
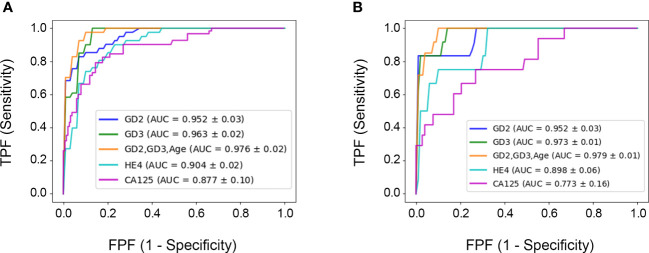
Receiver operator curves for each biomarker. **(A)** 5× cross-validation for the overall cohort. **(B)** 3× cross-validation for the segmented early-stage population. The ROC curve and diagnostic performance of GD2 and GD3 was done for all samples FIGO I–IV (n=41) or for segmented data FIGO stages I and II (n=14). The area under the curve (ROC–AUC) values for each test and the p-values of comparisons are listed in the caption and in **Table 2**.

For the overall population (FIGO stages I–IV, n=41) compared to the control (n=159), the model was validated using the 5× cross-validation method. GD2 (AUC, 0.952) and GD3 (AUC, 0.963) each independently performed significantly better than CA125 (AUC, 0.877) (GD2, p=0.032; GD3, p<0.001) (**Figure 4A**).

As an important subanalysis, the model cohort was segmented for the early-stage subset (FIGO stage I/II, n=14) versus controls (n=159), and the model was 3× cross-validated. In this early-stage subset, GD2 (AUC, 0.952) and GD3 (AUC, 0.973) each independently performed significantly better than CA125 (AUC, 0.773) (GD2, p=0.033; GD3, p=0.043) (**Figure 4B**).

The panel combining GD2+GD3+age (AUC, 0.976) was statistically superior to CA125 (AUC, 0.877) for the overall population of the model cohort (FIGO stages I–IV, respectively, p=0.003). Additionally, for the early-stage subset (FIGO stages I–II), the panel combining GD2+GD3+age (AUC, 0.979) was statistically superior to CA125 (AUC, 0.773; p=0.006). Including CA125 and/or HE4 in the GD2+GD3+age panel did not improve predictions (p=0.100); hence, they were omitted from the report. All the analyses are summarized in **Table 2**.

### 3.5 Sensitivity and specificity performance of the diagnostic algorithm

Metrics were compared at different thresholds of the ROC curve, setting the sensitivity at 97.6% for all models, and performance was compared to the clinically validated threshold of 35 U/ml for CA125.

In the overall population (FIGO I–IV), a combination of GD2+GD3+age (sensitivity of 97.6%) demonstrated superior sensitivity to CA125 (sensitivity of 63.4%) (p<0.001) with similar specificity (91.2% and 91.8%, respectively). In the early-stage subset (FIGO I–II), both GD2+GD3+age and CA125 had similar specificity (91.2% and 91.8%, respectively), but GD2+GD3+age had a sensitivity of 100%, while CA125 had lower sensitivity (57.1%). Including CA125 and/or HE4 to the panel of GD2+GD3+age did not improve predictions for either the overall cohort (FIGO I–IV) or the early-stage subset (FIGO I–II) populations; hence, they are omitted in the report.

## 4 Discussion

We detected consistent and statistically significant increases in expression of GD2 and GD3 in tissue and serum samples from individuals with OC, for all epithelial OC subtypes and FIGO stages, compared to controls. GD2 and GD3 levels were low in all non-OC samples, including healthy tissue, other cancers, and other non-cancerous gynecological conditions. GD2 and GD3 levels were significantly higher in invasive OC than in borderline (non-invasive) tumor samples.

Using quantitative data from the serum samples, we developed a diagnostic algorithm that included GD2+GD3+age, which is statistically superior in AUC to CA125 and HE4. Reportedly, tumor marker gangliosides can be found in plasma from cancer patients (20, 26), and GD3 expression in OC reportedly may play a role in ovarian cancer immune evasion (18). Our report advances the field, as it is the first study showing that quantification of gangliosides GD2 and GD3 and may be useful in the accurate diagnosis of OC.

### 4.1 GD2 and GD3 were elevated in solid tissues of ovarian cancer patients but not in healthy donors

GD2 and GD3 were detected in the tumor tissue of patients diagnosed with all subtypes of OC studied (clear cell, endometrioid, high-grade serous, low-grade serous, mucinous) and all OC FIGO stages, including early stages. Staining was homogeneously distributed in the plasma membrane and in cytoplasmic organelles, consistent with reports of the cellular distribution of GD2 and GD3 (27, 28). Furthermore, there was no effect of post-menopausal status (defined as age ≥50 years as per literature (29)) on GD2 and GD3 expression in both OC and borderline tumor samples.

Tissue block sections contained large tissue sections that included tumor tissue, and healthy surrounding tissue, e.g., fallopian tube, ovary, and endometrium, that can serve as internal control for the determination of GD2 and GD3 specificity. There was no GD2 and GD3 staining in any of the surrounding healthy tissues or in any other control tissue including fallopian tubes, indicating that GD2 and GD3 are highly selective for ovarian tumor tissue. In parallel, we conducted IHC studies for HE4. HE4 expression was not readily observed in mucinous tumors, or in 30% of stage I OC cases; furthermore, HE4 staining was detected in the normal fallopian tube tissue. These results are consistent with other reports for HE4 (30, 31) and indicate that GD2 and GD3 have higher sensitivity and specificity than HE4.

Tissues from HGSOC patients treated with debulking surgery (PDS) exhibited significantly greater scores and percent positivity for GD2 and GD3 compared to HGSOC patients also treated with chemotherapy (NACT) prior to debulking. It is unlikely that this difference is due to reduced tumor mass in HGSOC-NACT because the percentage of tumor cells in all cores was approximately 90%, regardless of treatment. Rather, we postulate that NACT may possibly reduce the overall density of GD2 or GD3 per cell in the tumor tissues. This hypothesis remains to be investigated.

In summary, GD2 and GD3 expression was detected in all OC subtypes (including mucinous) and all FIGO stages. Incorporating GD2 and GD3 immunostaining may be useful in pathological clinical practice. However, although IHC is informative, it is limited by methodological complications, including a low limit of detection, antigen loss, poor antigen stability or recovery, lack of tumor cells in the section, signal variation across laboratories, and subjective scoring. For these reasons, IHC is a qualitative assay and is rarely used for quantification. In contrast, ELISA is a sufficiently sensitive and quantitative assay where it is possible to establish a baseline that is statistically pre-determined, making it superior to IHC, and was therefore developed for quantification.

### 4.2 A serum-based ELISA quantifies elevated GD2 and GD3 in OC patients compared to healthy donors

We developed a quantitative ELISA to analyze expression of GD2 and GD3 in serum. We observed a statistically significant increase in GD2 and GD3 in OC serum samples compared to the control in both the discovery and model cohorts. Notably, the detection of GD2 and GD3 in serum by ELISA serum yielded higher AUCs than the commonly used biomarkers, CA125 and HE4. We developed an algorithm combining GD2+GD3+age that affords significant sensitivity and specificity for OC. This finding highlights the importance of evaluating multiple data points that include biomarkers and patient health information to increase diagnostic power.

The heterogeneity of ovarian cancer is a major obstacle in discovering novel biomarkers to aid early detection (32). The biomarkers CA125 (and HE4 in some jurisdictions) are typically used during the workup of patients with signs and symptoms of OC (4, 5, 12). Unfortunately, serum levels of these biomarkers often yield unclear results (31, 33). Elevated levels of CA125 are associated with a variety of common benign conditions including uterine leiomyomata, pelvic inflammatory disease, endometriosis, adenomyosis, pregnancy, and even menstruation. A normal CA125 measurement alone does not rule out OC in up to 50% of early-stage cancers and 20%–25% of advanced cancers and has an overall sensitivity and specificity of 80% and 82% (5, 34). Additionally, our results confirm the low specificity of HE4 as a single marker (30, 31). Other markers available today such as carcinoembryogenic antigen (CEA) and cancer antigen 19-9 (CA19-9) may sometimes be used in clinical practice but are not sufficiently sensitive or specific for OC.

Notably, the sensitivity of a combination of GD2+GD3+age for stages I and II (100%) was superior to the sensitivity of CA125 (57%) in the same samples while maintaining equally high specificity. This suggests that GD2 and GD3 may be highly useful in patients with OC and low CA125 levels. This is clinically relevant, as the low CA125 patients are typically harder to diagnose, and up to 50% of women in early-stage OC have normal CA125 levels (35). Indeed, CA125 as a single biomarker for OC may lead to misdiagnosis of OC due to its variability and its presence in many non-cancerous conditions.

Diagnostic algorithms are a unique strategy for improving sensitivity and specificity of diagnosis. Indeed, algorithms combining CA125+HE4 have demonstrated the greatest promise thus far (12, 36), although studies showed low specificity (37). Novel diagnostic algorithms using more sensitivity and specific biomarkers are needed to improve diagnosis accuracy and identification of early-stage OC. In our study, inclusion of CA125 and HE4 to the GD2+GD3+age panel did not improve OC detection. However, it is important to consider inclusion of other biomarkers and patient health information to increase diagnostic power.

Indeed, this is best exemplified by the performance of our algorithm combining GD2+GD3+age. Thus, other OC biomarkers that are currently in development may be considered in a panel, such as RNA (38, 39), DNA methylation (40–42), circulating tumor cells (CTCs), and circulating tumor DNA (ctDNA) (43–45), the Cu isotope, and markers within exosomes, GSTT1, FOLR1, ALDH1, and mRNAs, most likely in conjunction with CA125 (32). Successful development of such combinations would require compatibility with serum biomarkers and preferably be of low cost, allow rapid readouts, and optimally should be amenable to decentralized procedures such as the ELISA presented herein.

Aside from GD2 and GD3 being useful for us to exploit as biomarkers, we posit that the presence of GD2 and GD3 in serum suggests a biological role. Indeed, cell-bound GD2 and GD3 appear to be etiological to cancer onset or progression, lower the threshold for activation of receptor tyrosine kinases, and cause immune suppression allowing tumors to evade immune responses (14, 16, 17). Evidence that shed GD2 and GD3 are at least in part present in extracellular vesicles (ECVs) (46, 47) raises the possibility that horizontal transmission or tissue homing of GD2/3^+^–ECVs may play a role in these biological processes such as immune suppression.

Limitations of this study include the overall relatively small sample size, lack of ethnic diversity, and lack of diversity of benign ovarian tumors/other gynecological conditions that are likely to be represented in the intended target population. Additionally, tissue and blood samples were obtained from three different biobanks to obtain sufficient sample sizes, and not all samples were case controlled. It is possible that different curation practices introduced potential variations depending on the collection procedure, storage conditions, and sample quality. For example, we observed a quantitative difference in GD2 and GD3 levels between the discovery and model cohorts, which are likely attributed to differences in sample quality (the discovery cohort included older samples with an undocumented number of freeze/thaw cycles), while the model cohort contained high-quality case-controlled samples. Finally, menopausal status was not available from all biobanks. Although age is commonly used when menopausal status is unknown, future studies should limit samples to those with documented menopausal status. The introduction of bias was limited by blinding the pathologists who scored the IHC results and blinding ELISA operators to the clinical diagnosis of the samples. The computer modeling used cross-validation to controls for upward bias in estimating operating characteristics of the logistic regression model. Subsequent studies are needed to specifically address more cases of benign ovarian tumors and other gynecological conditions in larger more diverse populations to afford a reliable diagnostic for this unmet clinical need.

In summary, our results demonstrate that GD2 and GD3 are elevated both in tissue and serum samples of OC. Our diagnostic modeling indicates that GD2, GD3, and age are strong candidates for building a diagnostic panel for OC. Our results are a first proof of concept that quantification of GD2 and GD3 in serum afforded significantly better sensitivity than the currently used CA125 and HE4 OC biomarkers for diagnosis of multiple OC subtypes and for all OC stages, including early-stage diagnosis. The quantification of the tumor marker ganglioside family could prove useful for the detection or prognosis of many types of cancer, in a tissue-agnostic manner, from serum. A diagnostic panel could be used in symptomatic patients to facilitate rapid early determination of malignancy, accelerate intervention, and reduce the number of individuals undergoing invasive and expensive diagnostic procedures such as exploratory laparotomy. Validation of such a test could reduce patient wait time, expedite treatments and reduce mortality due to OC.

## Data availability statement

The original contributions presented in the study are included in the article/**Supplementary Material**. Further inquiries can be directed to the corresponding author.

## Ethics statement

The studies involving human participants were reviewed and approved by Institutional Review Boards at each biobank. TMAs, single biopsy tissue as paraffin-embedded blocks and blood samples were procured from biobank sources. Ethical approval included the Jewish General Hospital biobank, Montreal, QC, Canada (protocol #15-070), and the CRCHUM biobank, Montreal, QC, Canada (protocol #BD04.002). All ethical approval guidelines were followed. Informed consent was obtained from all individuals by the respective institution prior to specimen collection. The patients/participants provided their written informed consent to participate in this study.

## Author contributions

AG, AP and AN conceived, planned, designed, and carried out the experiments and prepared most figures and tables. AG, AP and AN (in order of contribution) curated, analyzed, and cross-validated their data. AG, EM and IH contributed to scoring IHC. JK, KL, AB, BC, EM, AY, EC, A-MM-M, and WG curated and provided clinical serum samples for ELISA. EM, AY and WG curated and provided tissues and tumor microarray samples for IHC. FB and WT generated and characterized the mAbs. KK, CG and AD conducted statistical analysis and the cross-validation. PG, SB and AJ contributed to the analyses and interpretation of the results and prepared some figures and tables. HS generated the concept, helped design experiments, helped with the analyses, and supervised the work. HS, AJ and AG lead the organization and writing of the manuscript, and the iterative editing of versions of the manuscript had contributions primarily by AP and by SB. All authors reviewed and approved the final version of the manuscript. AG, AP and AN share first authorship, in the order of their relative contributions. The manuscript has value only due to the cooperative and cross-validating nature of their contributions. Authors formally agreed to the order in which they are listed. All authors contributed to the article and approved the submitted version.
