# Supplementary material for: GD2 and GD3 gangliosides as diagnostic biomarkers for all stages and subtypes of epithelial ovarian cancer
Source: Front Oncol. 2023 Apr 14;13:1134763. doi: 10.3389/fonc.2023.1134763 (PMC10145910; doi:10.3389/fonc.2023.1134763)
Supplement: Supplementary file 1 [file DataSheet_1.pdf]

Supplementary Material

GD2 and GD3 gangliosides as diagnostic biomarkers for all stages and subtypes of epithelial ovarian cancer

Alba Galan [1#], Arturo Papaluca [1,2#], Ali Nejatie [1,2#], Emad Matanes [1,3], Fouad Brahimi [1], Wenyong Tong [1,2], Ibrahim Yaseen Hachim [4], Amber Yasmeen [3], Euridice Carmona [5], Kathleen Oros Klein [6,7], Sonja Billes [8], Ahmed E. Dawod [8], Prasad Gawande [8], Anna Milik Jeter [8], Anne-Marie Mes-Masson [5,9], Celia M.T. Greenwood [6,7], Walter H. Gotlieb [3], and H. Uri Saragovi [1,2,10 *].

authors share first authorship, in order of their relative contributions

*** Correspondence:**
H. Uri Saragovi
email: Uri.Saragovi@mcgill.ca

1 Supplementary Tables and Figures.

1.1 Supplementary Tables

Supplementary Table 1. Clinical Characteristics of Tissue Samples in Immunohistochemical Studies

Tissue Type	Subjects	% of total samples
**Single Biopsy Blocks**		
**Pathology Diagnosis**		
Invasive Epithelial Ovarian Carcinoma	24	19%
Borderline Epithelial Ovarian Carcinoma	7	7%
Controls	66	68%
OC-Adjacent Tissue Non-Cancer (Internal Control)	24	36%
Borderline-Adjacent Tissue Non-Cancer (Internal Control)	7	11%
Benign Gyn Condition Ovary	7	11%
Normal Ovary	12	18%
BRCA+	2	16%
Breast Cancer	5	42%
Healthy ovary from non-ovarian cancer	5	42%
Normal Fallopian Tube	1	2%
Normal Endometrial Tissue	15	23%
**Histopathological Subtype**		
High Grade Serous (HGSOC)	18	19%
Clear cell	3	1%
Endometrioid	3	1%
Mucinous	0	0%
**Primary Treatment (HGSOC)**		
PDS	10	56%
NACT	8	44%
**Tissue Microarrays (TMA)**		
**Pathology Diagnosis**		
Invasive Epithelial Ovarian Carcinoma	190	82%
Borderline Epithelial Ovarian Carcinoma	12	5%
Controls	31	13%
Benign Gyn Condition Ovary	4	13%
Normal Ovary	12	39%
Normal Fallopian Tube	8	26%
Tumor Adjacent Normal (Unknown Organ) Tissue	7	23%
**Histopathological Subtype**		
High Grade Serous (HGSOC)	102	55%
Clear cell	37	19%
Endometrioid	35	18%
Mucinous	16	8%
**Primary Treatment (HGSOC)**		
PDS	58	57%
NACT	44	43%

Supplemental Table 2: Clinical Characteristics of the Discovery Cohort

**Age, years (N=379)**		
Mean (SD)	60 (12.8)	
Median (range)	60 (23-99)	
**Race / Ethnicity (N=379)**	**Subjects**	**Percentage**
White	277	73%
Hispanic	15	4%
Black	3	1
Asian	2	1
Not Reported	132	35%
**Menopausal Status** (N=379)		
Pre (< 50 years)	67	18%
Post (≥ 50 years)	309	82%
Not Reported	3	1%
**Pathology Diagnosis** (N=379)		
Epithelial Ovarian Cancer	214	56%
Non-malignant Gyn Condition	72	19%
Healthy Donor	81	21%
Other Cancers	12	3%
**OC FIGO Stage (**N=214)		
I	66	31%
II	42	20%
III	83	39%
IV	23	11%
**OC Subtype (**N=214)		
High Grade Serous	111	52%
Clear cell	28	13%
Endometrioid	28	13%
Mucinous	4	2%
Low Grade Serous	3	1%
Borderline	40	19%
**Non-malignant Gyn Condition** (N=72)		
Cystadenoma	30	42%
Fibroma	25	35%
Endometrial Intraepithelial Neoplasia	7	10%
Lipoleiomyoma	3	4%
Not Reported	2	3%
Fibrothecoma	1	1%
Cystadenofibroma	1	1%
Chronic Endometritis	1	1%
Endometriosis	1	1%
BRCA+	1	1%
**Other Cancers (**N=12)		
Breast Cancer	6	50%
Endometrial Cancer	3	25%
Vaginal Cancer	1	8%
Thyroid Cancer	1	8%
Lymphoma	1	8%

Supplemental Table 3: Clinical Characteristics of the Model Cohort

**Age, years (N=200)**		
Mean (SD)<br>Median (range)	58 (12.8)<br>58 (18-86)	
**Race/Ethnicity (N=200)**	**Subjects**	**Percentage**
White	131	66%
Hispanic	52	26%
Black	9	5%
Asian	4	2%
American Indian	2	1%
Not Reported	2	1%
**Menopausal Status (N=200)**		
Pre (< 50 years)	33	17%
Post (≥ 50 years)	167	84%
**Pathology Diagnosis (N=200)**		
Epithelial ovarian cancer	41	21%
Non-malignant Gynecological Conditions	17	9%
Other Cancers	32	16%
Healthy Donors	110	55%
**FIGO Stage (n=41)**		
I	9	22%
II	5	12%
III	14	34%
IV	10	24%
Not Reported	3	7%
**OC Subtype (N=41)**		
High Grade Serous	21	51%
Clear cell	3	7%
Endometrioid	2	5%
Granulosa cell	1	2%
Not Reported	14	34%
**Non-malignant Gyn Conditions (N=17)**		
Dysfunctional Uterine Bleeding	1	6%
Pelvic Inflammatory Disease	1	6%
Uterine Fibroids	2	12%
Polycystic Ovary Syndrome (PCOS)	6	35%
Endometriosis	7	41%
**Other Cancers (N=32)**		
Breast Cancer	5	16%
Cervical Cancer	3	9%
Non-Small Cell Lung Cancer	4	13%
Colon Cancer	5	16%
Bladder Cancer	5	16%
Lymphoma	5	16%
Leukemia	5	16%

1.2 Supplementary Figures

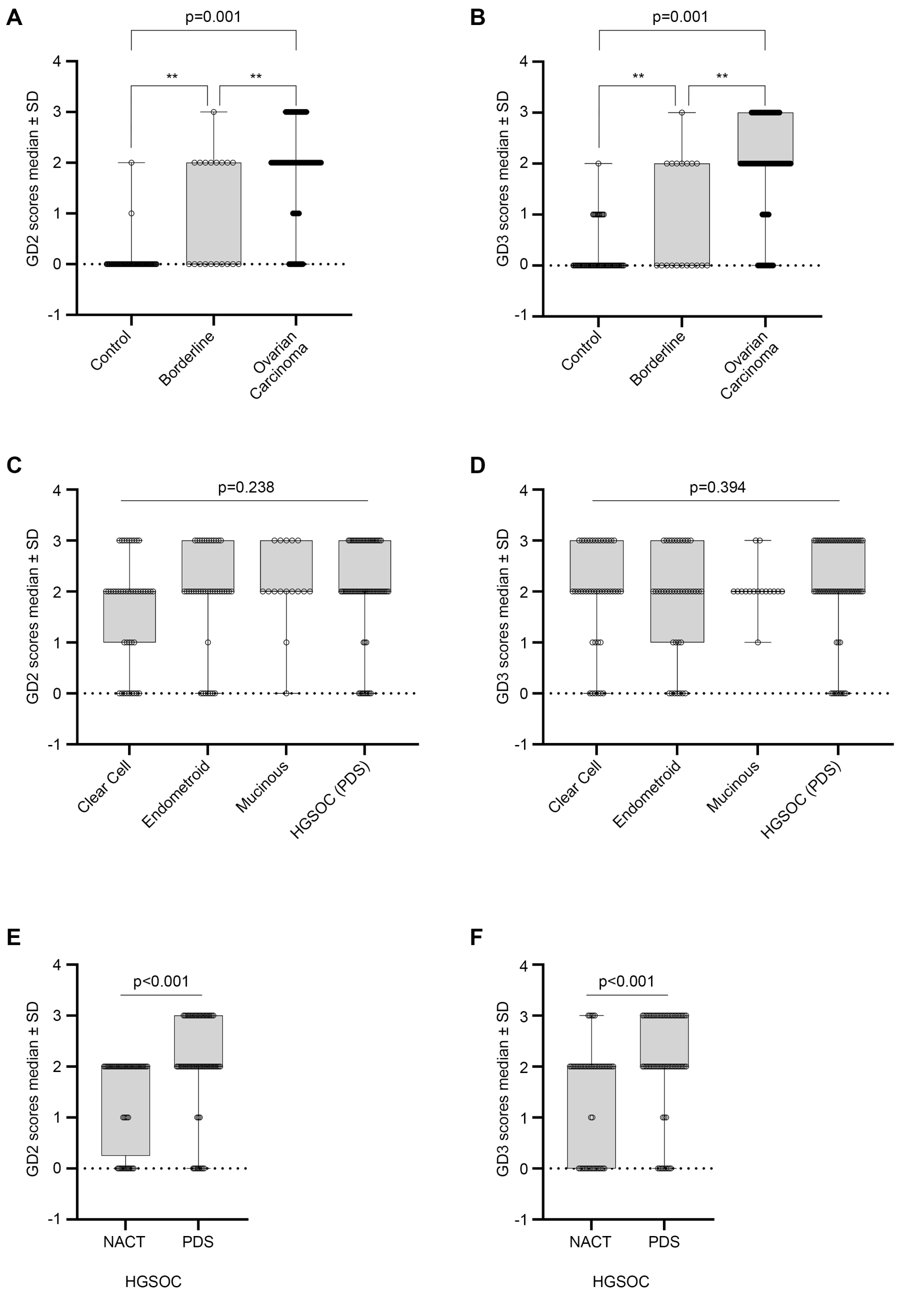

Supplemental Figure 1. Distribution of GD2 and GD3 staining from tissue immunohistochemistry. (**A, B**) Distribution of GD2 and GD3 scores in normal, borderline, and OC patients. (**C, D**) Distribution of GD2 and GD3 scores in OC subtypes. (**E, F**) Distribution of GD2 and GD3 scores in high grade serous OC (HGSOC) patients treated with primary-debulking surgery (PDS) or neoadjuvant therapy (NACT). Samples were classified according to their intensity: no immunoreactivity (0), 1+ (weak stain), 2+ (stain), 3+ (strong stain). Data are shown as the arithmetic average of GD2 or GD3 scores ± standard deviation (SD), and max/min values. Dots represent individual values.

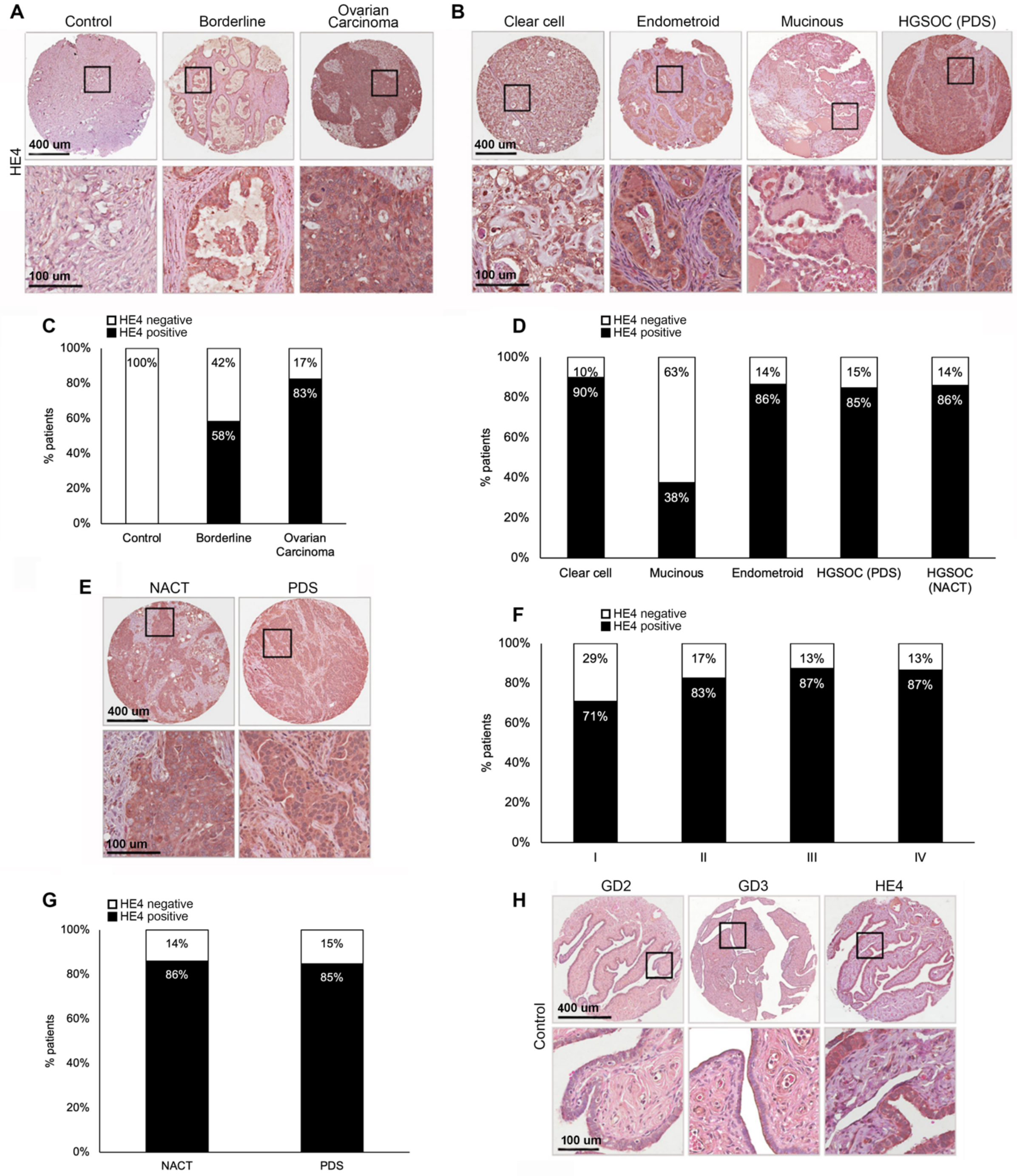

Supplementary Figure 2: HE4 is highly expressed in tissues of different OC subtypes and FIGO stages, including HGSOC treated with neoadjuvant chemotherapy (NACT). Representative images showing HE4 immunohistochemistry in (**A**) normal control ovary, borderline, and ovarian carcinoma tissue biopsies or (**B**) in different OC subtypes. The bottom panels show a higher magnification of tissue within the black boxes (scale bars indicated). (**C**) Percentage of HE4 in normal control ovary, borderline, and OC groups. (**D**) Percentage of HE4 in OC tissue subtypes. Mucinous subtype had significantly lower HE4 expression than the other invasive subtypes. Mucinous was reduced versus HGSOC ($p<0.001$), Clear Cell ($p<0.001$), and Endometrioid

(p<0.001). Scores were considered negative (“0” and “1”) or positive (“2” and “3”). OC (n=195) subtypes consisted of HGSOC (n=102, including PDS, n=59 and NACT, n=43), Clear cell (n=40), Endometroid (n=37), Mucinous (n=16), and borderline (n=12). Control (n=24) consisted of healthy ovaries (n=14), benign gynecological conditions (n=7), normal fallopian tubes (n=2), and normal tissue adjacent to ovarian cancer (organ not documented, n=1). (**E**) Representative images showing HE4 expression in patients treated with neoadjuvant chemotherapy (NACT, n=43) or with primary-debulking surgery (PDS, n=58). The bottom panels show a higher magnification of tissue within the black boxes (scale bars indicated). (**F**) Quantification of HE4 expression by FIGO stages shows significantly lower HE4 expression in Stage I (p=0.030). (**G**) Quantification showed no significant differences in HE4 expression by treatment (p=0.500). (**H**) Representative images showing GD2, GD3 and HE4 immunohistochemistry of fallopian tube biopsies from non-cancer control patients. The bottom panels show a higher magnification of tissue within the black boxes (scale bars indicated). HE4, but not GD2 or GD3, was expressed in fallopian tubes of healthy patients.

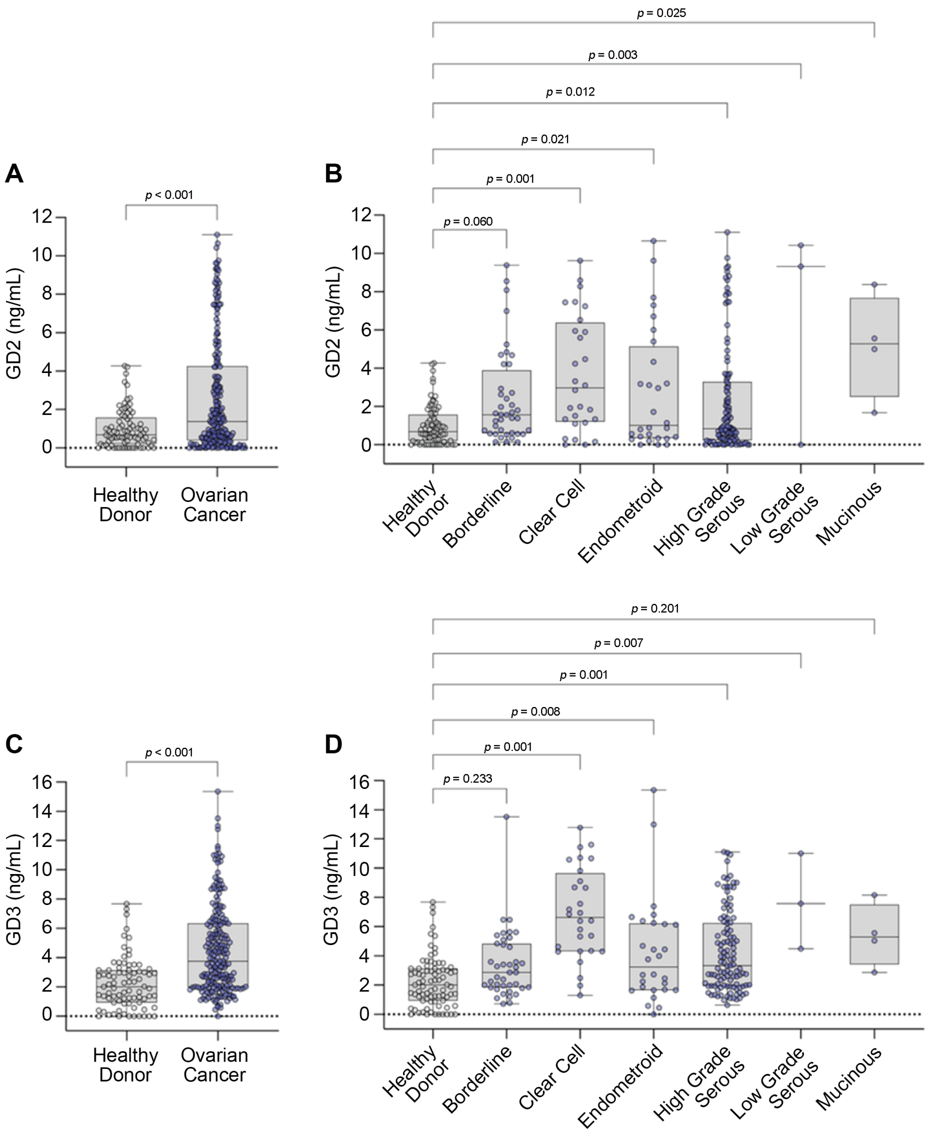

Supplemental Figure 3: Analyses of GD2 and GD3 in liquid biopsies by OC subtype. (A, C) Box plots displaying concentrations GD2 and GD3 in healthy donors (n=81 patients) and OC patients (n=214 patients). (**B, D**) Box plots displaying concentrations of GD2 and GD3 in healthy donors (n=81) borderline ovarian tumor tissue (n=40), and OC tissue by cancer subtype: Clear cell (n=28), Endometrioid (n=28), High grade serous (n=111), Low grade serous (n=3), Mucinous (n=4). Box plots show median, upper and lower quartiles, and max/min values. Dots represent individual values.

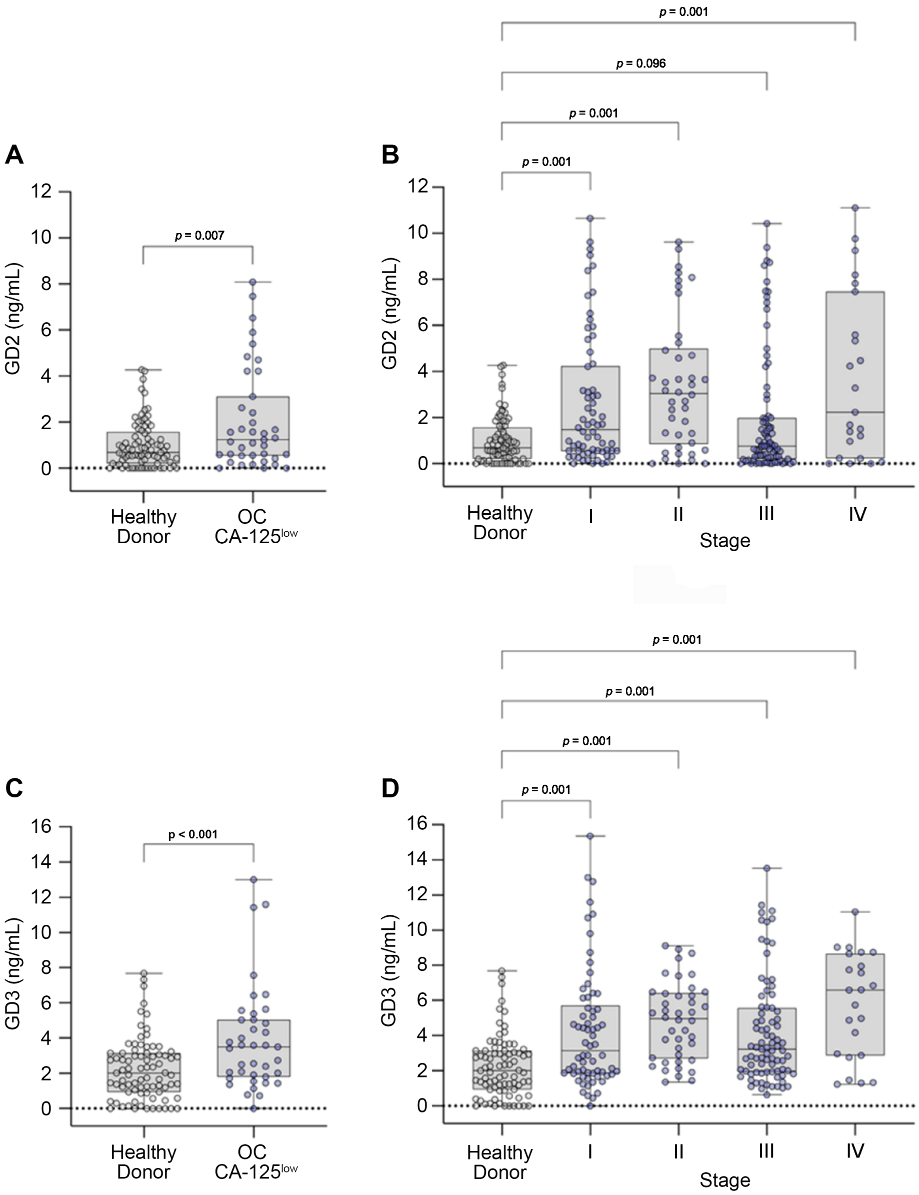

Supplemental Figure 4. Analyses of GD2 and GD3 in liquid biopsies by OC stage and in OC patients with low CA125 expression. (**A, C**) Box plots displaying concentrations of GD2 or GD3 in healthy donors (n=81 patients) and OC patients with low CA125 expression, defined as CA125 <35 U/mL (n=39 patients). (**B, D**) Box plots displaying concentrations of GD2 or GD3 in healthy donors and OC patients by FIGO Stage: Stage I (n=66), Stage II (n=42), Stage 3 (n=83), Stage 4 (n= 23). Box plots show median, upper and lower quartiles, and max/min values. Dots represent individual values.

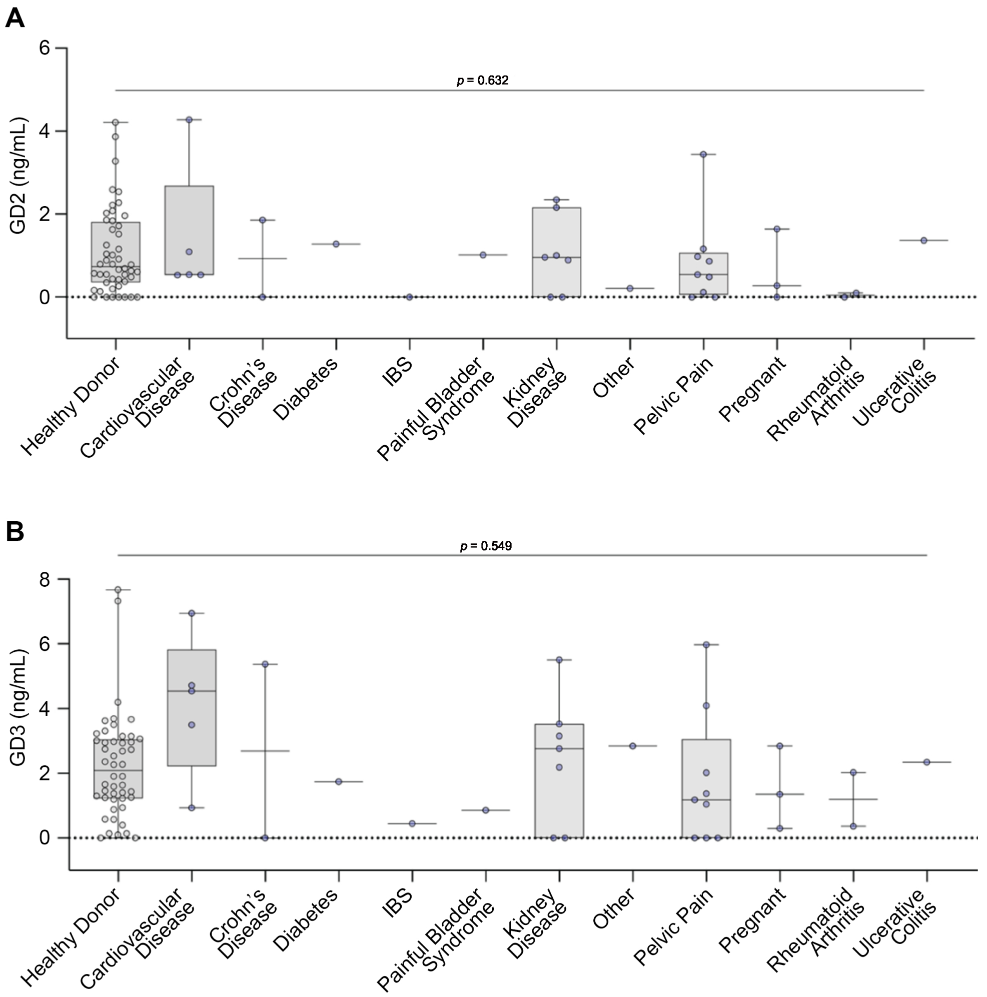

Supplemental Figure 5. Analyses of GD2 and GD3 in liquid biopsies comparing normal healthy versus patients with other conditions. Box plots displaying concentrations of (**A**) GD2 and (**B**) GD3 in healthy donors (n=48) and patients with different conditions: Cardiovascular disease (n=5), Crohn's disease (n=2), Diabetes (n=1); Irritable bowel syndrome (n=1); Painful bladder syndrome (n=1), Kidney disease (n=7), Other (n=1), Pelvic pain (n=9), Pregnancy (n=3), Rheumatoid arthritis (n=2), Ulcerative colitis (n=1). Box plots show median, upper and lower quartiles, and max/min values. Dots represent individual values.

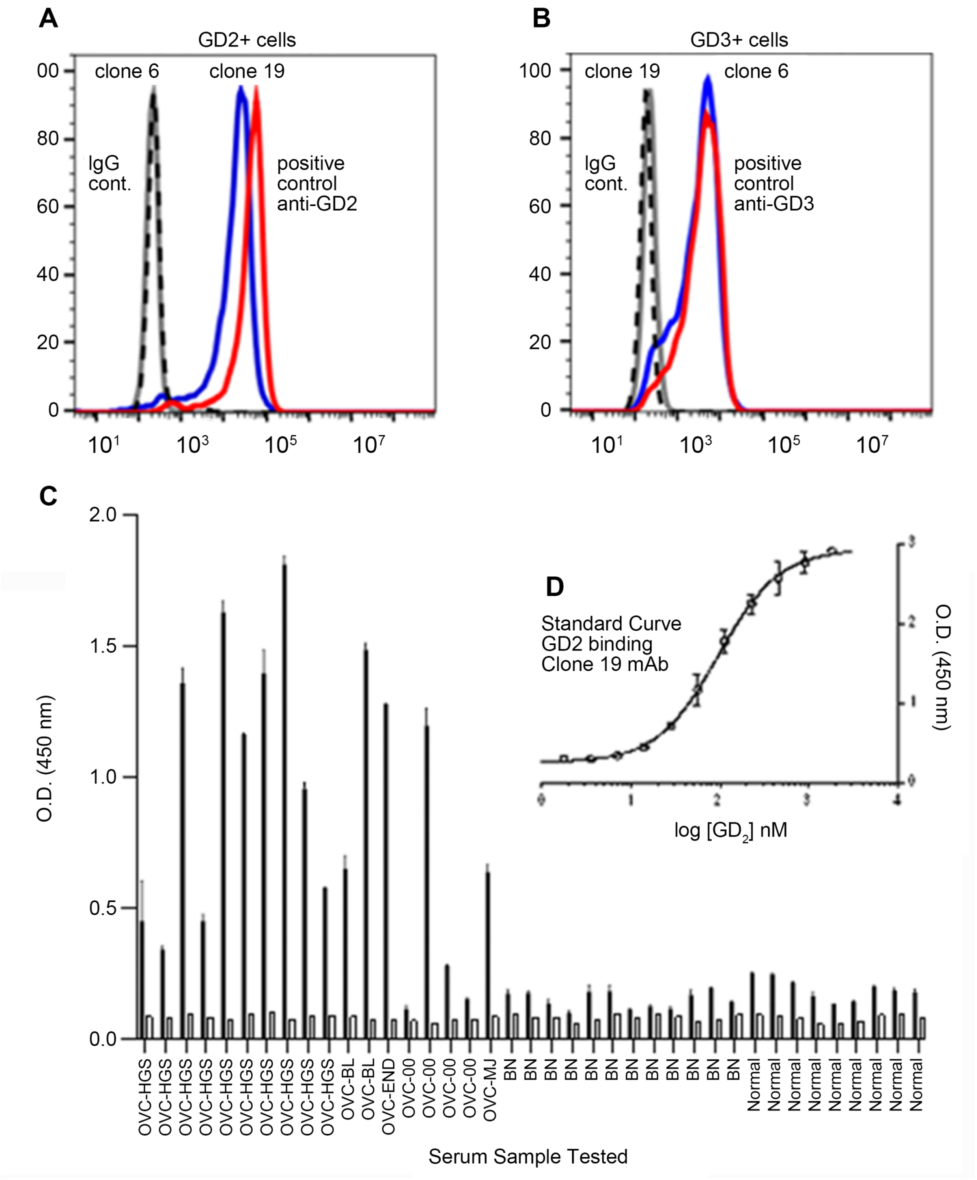

Supplemental Figure 6: Characterization of primary mAbs by flow cytometry and ELISA. Binding assays were done by quantitative flow cytometry using EL4-GD2$^+$ (expressing GD2 but not expressing GD3) or EL4-GD3$^+$ cells (expressing GD3 but not expressing GD2) (see Methods and (1)). Histograms represent the mean channel fluorescence. (**A**) Anti-GD2 mAb clone 19 (blue) binds specifically to cells expressing GD2 (but not expressing GD3). Positive control is anti-GD2 (red, commercial source). Negative controls are mouse IgG (dashes) and anti-GD3 mAb clone 6 (gray). (**B**) Anti-GD2 mAb clone 6 (blue) binds specifically to cells expressing GD3 (but not expressing GD2). Positive control is anti-GD3 (red, commercial source, methods described below). Negative controls are mouse IgG (dotted) and anti-GD2 mAb clone 19 (gray). (**C**) Example of alternative readout for ELISA, using biotinylated anti-GD2 mAb clone 19 followed by streptavidin conjugated to-HRP (Methods described below). Shown is the raw Optical Density data for the indicated human serum samples. O.D, 450 values for test samples using primary biotinylated anti-GD2 and secondary streptavidin-HRP (black) and for controls using only secondary streptavidin-HRP (white). (**D**) GD2 standard curve is shown (r^2=0.9948). The limit of detection is ≤10 nM GD2. Assays were performed blinded by two operators.

In an alternative ELISA method, the in house mAbs were biotinylated using a commercial kit (Thermo Scientific, Cat. 21900) (2) and detection used streptavidin conjugated with HRP (Thermo Scientific, Cat. 21130, used at 1:20,000). The biotin-streptavidin ELISA method followed the protocol described in the Methods with the exception that primary mAbs were biotinylated. Overall both ELISA methods yielded equivalent results, but we noted that some mAbs can exhibit batch-to-batch variability, which requires re-optimization of the assay on an ongoing basis.

2 Supplementary References

1. Tong W, Maira M, Roychoudhury R, Galan A, Brahimi F, Gilbert M, et al. Vaccination with Tumor-Ganglioside Glycomimetics Activates a Selective Immunity that Affords Cancer Therapy. Cell Chem Biol. 2019;26(7):1013-26 e4.

2. Brahimi F, Maira M, Barcelona PF, Galan A, Aboulkassim T, Teske K, et al. The Paradoxical Signals of Two TrkC Receptor Isoforms Supports a Rationale for Novel Therapeutic Strategies in ALS. PLoS One. 2016;11(10):e0162307.
